# Emergency surgery preoperative delays: realities, economic impacts and gains of a second emergency operating theatre

**DOI:** 10.1308/rcsann.2024.0021

**Published:** 2024-04-02

**Authors:** Á Lucey, S Beecher, R McLaughlin

**Affiliations:** Department of General Surgery, Galway University Hospital, Ireland

**Keywords:** Emergency theatre, Time-to-theatre, Cost, Kaizen

## Abstract

**Introduction:**

Time-to-theatre (TTT) is a key performance indicator of theatre efficiency and delayed TTT incurs significant costs and poor clinical outcomes. An increasing Irish population in conjunction with an ageing population puts increasing pressure on emergency surgical services across Ireland. We examined our institution's experience with introducing a second emergency theatre and semi-elective theatre lists for acute surgical patients.

**Methods:**

A retrospective review of electronic, prospectively maintained databases was performed between 1 February 2018 and 31 January 2020. A cost analysis was conducted to assess the economic impact of delayed TTT. The cost-saving benefit of introducing a second emergency theatre and semi-elective Kaizen lists was then calculated and compared with 2012–2014 figures from our institution.

**Results:**

In total, 6,679 procedures were performed. Overall mean TTT was 16h, 10h shorter than before the introduction of a second emergency theatre and Kaizen theatre lists (*p* < 0.001). Patients aged >65 years, who are historically a significantly disadvantaged group, had a shorter TTT following the introduction of a second emergency theatre. The economic advantage of a second emergency theatre resulted in a cost saving of **€**3,674,538 over 24 months.

**Conclusion:**

Investment in emergency surgical services resulted in more efficient access to emergency theatres. There was a reduction in out-of-hours operating across all specialties and across the more at-risk groups such as those over the age of 65, who had an overall reduction in TTT. This had significant financial benefits and likely reduced the clinical risk associated with delayed TTT and out-of-hours operating.

## Introduction

University College Hospital Galway (UCHG) is a tertiary referral centre serving a catchment area with a population in the region of 1,000,000, an increase of approximately 33% in the past 6 years.^1^ UCHG is a model 4 hospital providing 24/7 care in acute surgery along with elective surgery across a wide number of specialties including general, orthopaedic, plastic, urological, cardiothoracic, vascular, maxillofacial, ear nose and throat, and ophthalmic surgery.^2^ In 2021, UHCG had 68,887 emergency department attendances, an increase of 8.3% on 2020 figures.^3^

In 2014, O’Leary *et al* examined UCHG’s experience with running a designated emergency theatre for acute surgical patients, using time-to-theatre (TTT) as a key performance indicator, and subsequently performing a cost analysis of the economic impact of TTT delays.^1^ With orthopaedics remaining the exception, running a parallel emergency orthopaedic theatre, the emergency theatres in our institution are used by all surgical specialties. There is some additional capacity in the other theatres when elective theatre lists finish early and staffing allows. Following increased demands on the UCHG emergency theatre, a second emergency theatre was introduced in July 2017 to increase access and reduce TTT for acute surgery patients, and to reduce the amount of out-of-hours emergency surgery being conducted, where possible. Along with a second emergency theatre, dedicated Kaizen lists were also introduced in July 2017 for surgical specialities dealing with a high volume of acute surgical emergencies. The specialities utilising Kaizen lists include plastic surgery, urology, ear nose and throat, and maxillofacial surgery. Kaizen, the Japanese word for continuous improvement, is a compound of two Japanese words.^4–6^ ‘Kai’ (meaning ‘change’) and ‘zen’ (meaning ‘good’) together translate as ‘good change’ or ‘improvement’.^7^ The Kaizen list attempts to facilitate acute surgical emergencies that are not suitable to wait for an elective surgical list but can be facilitated on a semi-elective, semi-emergent basis. Urology, plastic surgery, ear, nose and throat, and maxillofacial surgery treat a very high volume of acute surgical emergencies and often have extended hospital stays because of the limited availability of the emergency theatre. The introduction of a second theatre and the Kaizen lists were intended to reduce pressures on the emergency theatre and, in turn, reduce the length of stay of the acute surgical patient cohort.

The primary aim of our study is to review TTT for acute surgical emergencies as a key performance indicator following the introduction of a second emergency theatre and semi-elective Kaizen lists. The secondary aim is to perform a cost analysis to demonstrate whether there has been a reduced economic burden following reduced TTT delays with the introduction of a second emergency theatre and semi-elective Kaizen lists. Our primary objective was to determine whether a second emergency theatre was economically beneficial as well as contributing to better patient care.

## Methods

We performed a single-centre, retrospective cohort study that involved a review of a prospectively maintained electronic theatre database over 24 months between 1 February 2018 and 31 January 2020, 6 years following the initial review by O Leary *et al* and following the introduction of a second emergency theatre and Kaizen semi-elective emergency surgery lists.^1^ The electronic database has a priority categorisation system that divides cases into high, medium or low priority. High-priority cases included any immediately life-threatening cases that needed surgical intervention within 30min. Any child under the age of 12 requiring surgery was also categorised as a high priority. Medium-priority cases were those that needed an operation within 6h and low-priority cases were those that needed an operation within 24h.^1^ Given that the Kaizen list is carried out in the emergency theatre, patients whose operations were scheduled on these semi-elective lists are also reflected in this electronic emergency theatre database.

The data parameters included patient sex, patient age, operation, specialty, time added to the list, operation start time and operation finish time. The ‘time added list’ represents the time the decision was made that a patient requires surgery. The ‘operation start time’ was a separate parameter and indicated the time the patient arrived in the theatre. TTT represents the period between the ‘time added to list’ and the ‘operation start time’. Working hours were defined by an operation commencement time between 9am and 5pm on any given day, including weekend days. Out-of-hours operating was assessed based on the operation start time, outside the hours of 9am and 5pm.

Costs were calculated according to the hospital cost of an overnight bed, €1477 per night.^1^ This excludes the cost of the procedure itself. The definition of delay was a TTT of more than 24h, representing a greater than 24h wait time for surgery. Further data analysis was performed in the <16 years age group and >65 years age group because both pose unique challenges that may influence TTT. The percentage of operations performed outside working hours was also calculated. The data were then compared with figures reported before the introduction of the second emergency theatre and Kaizen lists. Time is calculated in hours and minutes.

Statistical analysis was performed using SPSS version 27. Independent samples *t*-test was used to analyse mean TTT differences across the different time frames: 2012–2014 before the introduction of the Kaizen lists; and 2018–2020 following the introduction of the Kaizen lists. Pearson’s chi-squared test was used to analyse whether there was a relationship between operations occurring out of hours and a TTT of >24h.

The results were then compared with the 2016 data published by O’Leary *et al*. The domains that were directly compared included overall TTT and TTT per speciality, TTT in extremes of ages such as <16 and >65, the total number of procedures performed out of hours and finally the cost-saving benefit following the introduction of a second emergency theatre.

## Results

A total of 6,679 procedures were performed in the 24-month period analysed. Patient demographics are outlined in Table 1 and are further broken down by speciality. The overall mean age was 46 years and the male to female ratio was 1.7:1. In total, 18.7% of patients were <16 years of age, compared with 17.6% in the 2012––2014 data.^1^ Some 31.2% were >65 years of age, compared with 27.6% in the 2012–2014 data.

**Table 1:** Patient demographics

	Overall		General		Urology		Plastics		Vascular	
	2012–2014	2018–2020	2012–2014	2018–2020	2012–2014	2018–2020	2012–2014	2018–2020	2012–2014	2018–2020
Procedures, *n*	7,041	6,679	2,949	2,228	1,050	1,356	1,687	1,387	290	583
Age, years^a^ (range)	45 (0–98)	49 (0–97)	43 (0–96)	49 (0–94)	58 (0–96)	60 (1–95)	27 (0–95)	23 (0–94)	70 (13–98)	71 (17–95)
Male, *n* (%)	4,243 (60)	4,196 (63)	1,487 (50)	1,137 (51)	803 (76)	1,046 (77)	1,129 (67)	925 (66)	173 (66)	404 (69)
Female, *n* (%)	2,798 (40)	2,483 (37)	1,462 (50)	1,091 (49)	247 (24)	310 (22)	558 (33)	462 (33)	117 (34)	179 (31)
<16 years, *n* (%)	1,352 (17.6)	1,249 (18.7)	533 (18)	396 (17)	64 (6)	86 (6)	625 (37)	586 (42)	1 (<1)	0 (0)
>65 years, *n* (%)	1,943 (27.6)	2,088 (31.2)	772 (26)	569 (25)	433 (41)	589 (43)	209 (12)	173 (12)	203 (70)	405 (69)

^a^Median.

Table 2 details the overall mean TTT and mean TTT per speciality. The overall mean TTT was 16h 0min in the overall group compared with 26h 2min in the 2012–2014 data. This shows a 10h reduction in TTT (*p* < 0.001). The general surgery mean TTT was 11h 29min compared with 23h 23min in the 2012–2014 data. This shows an 11h 54min reduction in TTT across all general surgery cases (*p* < 0.001). The urology mean TTT was 25h 44min compared with 43h 56min in the 2012–2014 data. This shows an 18h 12min reduction in TTT across all urology cases, the biggest reduction in TTT across all groups (*p* < 0.001). The plastics mean TTT was the shortest at 11h 53min compared with 20h 8min in the 2012–2014 data. This shows an 8h 15min reduction in TTT across all plastic surgery cases (*p* = 0.005). The vascular mean TTT was 21h 26min compared with 37h 9min. This shows a 15h 43min reduction in TTT across all vascular surgery cases (*p* < 0.001). Pearson’s chi-squared test concludes that there is a statistically significant relationship between operations occurring outside normal operating hours and TTT >24h (*p* < 0.001).

**Table 2:** Mean time to theatre including overall and per specialty

	Overall		General		Urology		Plastics		Vascular	
Time-to-theatre (h:min)	2012–2014	2018–2020	2012–2014	2018–2020	2012–2014	2018–2020	2012–2014	2018–2020	2012–2014	2018–2020
All age groups	26:02	16:00	23:23	11:29	43:56	25:44	20:08	11:53	37:09	21:26
<16 years	10:36	06:31	12:15	07:12	02:46	04:13	10:06	05:05	12:40	N/A^a^
>65 years	36:52	21:10	32:12	13:19	53:52	30:29	40:56	21:36	37:25	21:55

^a^ N/A = Not applicable, as no vascular surgery was performed in patients under the age of 16.

The <16 years age group had a shorter mean TTT of 6h 31min when compared with 2012–2014, where the mean TTT was 10h 36min (*p* < 0.001). The >65 years age group had a mean TTT of 21h 10min which was 5h 10min or 32% longer than the overall mean TTT. The TTT in the >65 years age group improved when compared with 2012–2014, when the mean TTT was 36h 53min (*p* < 0.001).

Of the total of 6,641 procedures, 1,220 (18%) were performed out of hours. This is 7% lower than in 2012–2014, when 1,756 (25%) procedures were being performed outside the hours of 9am and 5pm. All of the surgical specialities exhibited decreased out-of-hours operating following the addition of the second emergency theatre and Kaizen lists (Table 3).^8^

**Table 3:** Surgical activity in the emergency theatre

	2012–2014	2018–2020	2012–2014	2018–2020	2012–2014	2018–2020	2012–2014	2018–2020	2012–2014	2018-2020
	Overall	Overall	General	General	Urology	Urology	Plastics	Plastics	Vascular	Vascular
Emergency operations, *n*	7,041	6,641	2,949	2,228	1,050	1,356	1,687	1,387	290	583
Operations out of hours, *n* (%)	1,756 (25)	1,220 (18)	910 (31)	556 (25)	221 (21)	204 (15)	191 (11)	62 (4)	106 (37)	138 (24)

Having demonstrated improvements in mean TTT with the addition of the second emergency theatre and Kaizen lists, we next wished to calculate the economic benefit of reduced TTT. Table 4 details where the additional costs from delays in TTT arise and shows the reduced cost associated with decreased length of stay related to shortened TTT. In total, a figure of €3,441,887 is generated from delayed TTT over 24 months, a reduction of >50% when compared with the €7,116,425 additional cost generated from delayed TTT in the 2012–2014 data.^1^ This reflects a saving of €3,674,538 following the introduction of the second emergency theatre and Kaizen lists, which reduced TTT across all specialities.

**Table 4:** Costs incurred from delayed time-to-theatre

	No. of patients			Cost (€)		
Hours	2012–2014	2018–2020	Extra nights	2012–2014	2018–2020	Difference (€)
24–48	1,342	905	1	1,983,435	1,335,685	647,750
48–72	531	348	2	1,569,604	1,027,992	541,612
72–96	241	87	3	1,112,911	385,497	727,414
96–120	129	46	4	762,632	271,768	490,864
>120	148	40	>5	1,687,841	420,945	1,266,896
Total				7,116,425	3,441,887	3,674,538

## Discussion

Out-of-hours surgery and delayed TTT are associated with increased morbidity and mortality, and can be used as surrogate key performance indicators of quality of care.^1^ The introduction of a second emergency theatre has enabled improved quality care for acute surgical patients at our hospital.

There are ongoing issues surrounding the provision of this type of service that need to be addressed, many of which are highlighted in this study. In the first instance, this study displays the ongoing high levels of acute surgical admissions in our hospital across 24 months. There are often resource allocation conflicts between elective and emergency surgery and this study highlights how the allocation of additional resources to emergency surgery can have both economic and patient safety-related benefits. This study demonstrates that the allocation of additional resources to the acute surgical service in the form of access to a second emergency theatre and Kaizen operating lists reduced the number of patients whose TTT was >24h from 34% to 21%.^1^ There was a significant reduction in mean TTT across all surgical specialties following the introduction of a second emergency theatre and Kaizen lists. Not only did the second emergency theatre directly benefit the specialties that were allocated additional Kaizen lists, but it also indirectly benefited the remaining specialities, such as general surgery, which now had increased access to emergency theatre.

The number of patients over the age of 65 requiring emergency surgery has increased from 1,943 patients in 2012–2014 to 2,088 patients in 2018–2020 cohort. This reflects a 7.3% increase in the number of patients over the age of 65 requiring emergency surgery between the two patient cohorts. Some 31.2% of the total patient cohort in 2018–2020 were over the age of 65, an increase of 3.6% when compared with 2012–2014 figures when 27.6% of the total patient cohort requiring emergency surgery were aged 65 and over. In 2014, the over-65 group represented 12.6% of the total population with this figure increasing to 14.4% by 2020. The Central Statistics Office has predicted that the population of Ireland will increase to 5.6 million by 2031, with those aged over 65 accounting for 17.9% of the total population of Ireland. By 2051 it is predicted that the population over-65 will represent 19.8%, almost one-fifth of the total.^9^ An ageing population puts increased demands on a healthcare system and will naturally result in greater numbers of elderly people requiring emergency surgery. The addition of a second emergency theatre had a significant bearing on the trends seen in TTT across the different age categories. Naturally, given the difficulties associated with comorbidities that come with increasing age, the over-65 category will experience an expected delay in TTT to allow for preoperative optimisation, where possible.^1^ It is, however, important that elderly populations are not deprioritised when it comes to emergency surgery.^10^ The addition of a second emergency theatre has decreased the mean TTT in the over 65 age group from 36h 52min to 21h 10min, which will undoubtedly have improved overall outcomes in this patient cohort. Unsurprisingly, the under-16 age group had the quickest mean TTT, particularly in the urology group where 85% of cases represented scrotal explorations, a highly time-sensitive procedure. The mean TTT in scrotal exploration across all ages was 2h 09min, well within the international recommendation of <4h, with 89% of cases meeting this target.^11,12^

The cost-saving benefits of a second emergency theatre were quite significant. It is important to note that additional overnight bed costs were only calculated following the first 24h TTT delay period, which may represent an underestimation of the final overall cost. Naturally, the running of an additional theatre required additional nursing, anaesthetic and ancillary personnel, which incurred additional costs; however, the infrastructure was already in place in the hospital with the availability of an empty theatre. It must also be considered that, regardless of the timing of the procedure, there will always be a cost of the procedure itself, including equipment and indirect costs such as lighting and heating.^1^ It is likely that the money invested in improving the resources available to acute surgical services through the addition of a second emergency theatre has been recouped in the savings made through reduced TTT and overall length of stay.

The estimated cost of running an operating theatre is approximately €560 per hour.^13^ Using this figure it can be estimated that if a second emergency theatre were operational for 39h per week, during the 24-month period this study was conducted, it would cost €2,271,360. When this additional cost of running a second operating theatre is considered and deducted from the estimated cost saved of €3,674,538, we can still deduce that a second emergency theatre can save up to €1,403,178 in a 24-month period, or over €700,000 per annum.

### Study limitations

Our study is not without its limitations. One limitation is the retrospective design of the study. It is also based on data from a single institution, and it would be interesting to compare the logistics of acute surgical services in other hospitals across the country and the cost-effectiveness of their model. To our knowledge, this study is the first to highlight the advantages of a Kaizen semi-elective, semi-emergent theatre list, and the economic and patient safety-related benefits it can have. A second limitation of our study is that it only addresses one patient outcome that has occurred as a result of short TTT (length of stay). We have not addressed any other potential comparative outcomes to further substantiate the improved quality of care that likely occurred following the introduction of a second emergency theatre, reduced TTT and shorter hospital stays for these patients. There is scope for further research in this area. Another limitation of our study is the fact that there are many other caveats involved in delayed TTT aside from theatre availability. Oftentimes, comorbid patients may need preoperative optimisation to make the operation safer; they may have outstanding investigations, often radiological in nature; or they might simply be awaiting an available postoperative bed to recover following their surgery. This needs to be considered as when these caveats occur, it does not simply imply a failure in the system but rather a multifactorial process. A final limitation of our study to be addressed is that we have not examined whether reduced TTT and shorter hospital stays following the introduction of a second emergency theatre has led to reduced postoperative complications, reduced reoperation and reduced readmission rates. This is another area in which there is scope for further research.

## Conclusions

The introduction of a second emergency theatre and Kaizen semi-elective theatre lists significantly reduced the number of patients waiting for emergency surgery for longer than 24h. Before the introduction of Kaizen theatre lists, 34% of patients (2,391/7,041) waited >24h for their surgery. This figure was reduced to 21% (1,426/6,679) in the 2018–2020 patient cohort following the introduction of a second emergency theatre and the Kaizen semi-elective emergency theatre. A second emergency theatre reduced TTT for the elderly population, a group that will be increasingly represented in the acute surgical patient cohort as our ageing population numbers increase. The number of emergency operations that took place outside the hours of 9am and 5pm (out of hours) was also reduced following the introduction of a second emergency theatre. Although not directly examined in this study, the cost incurred through the investment in acute surgical services will likely be largely outweighed by the cost-saving benefits associated with the overall reduced length of hospital stay in acute surgical patients.
